# Polymorphous Adenocarcinoma: A Rare Case Report with Unique Radiographic Appearance on CBCT

**DOI:** 10.1155/2021/8853649

**Published:** 2021-03-24

**Authors:** Jagadish Chandra, Junaid Ahmed, K. M. Veena, M. Vijayakumar, Nandita Shenoy, Nanditha Sujir

**Affiliations:** ^1^Dept. of Oral Surgery, Yenepoya Dental College, Yenepoya University, Deralakatte, India; ^2^Dept. of Oral Medicine and Radiology, Manipal College of Dental Sciences, Mangalore, Manipal Academy of Higher Education (MAHE), Manipal, Karnataka 576104, India; ^3^Dept. of Oral Medicine and Radiology, Yenepoya Dental College, Yenepoya University, Deralakatte, Karnataka, India; ^4^Department of Surgical Oncology, Yenepoya Medical College, Yenepoya University, Deralakatte, Mangalore, India

## Abstract

**Background:**

Polymorphous low-grade adenocarcinoma (PLGA) is a slow growing malignant tumor of minor salivary glands and is generally of indolent nature. However, according to the most recent WHO Classification of Salivary Gland Tumors (2017), the cancer is classified as Polymorphous AdenoCarcinoma (PAC). PAC presents as a less aggressive tumor, though it could on rare occasions demonstrate distant metastasis. *Case Presentation*. A 47-year-old man who was referred by a private practitioner for a CBCT scan in reference to a proliferative soft-tissue growth in the hard palate. The growth was mild and tender and there was Grade III mobility in relation to all the maxillary teeth. Panoramic radiograph taken previously had revealed evidence of alveolar bone loss in relation to the maxillary teeth and was inconclusive of any other findings. The CBCT scan revealed evidence of moth-eaten appearance of maxilla with destruction of medial and lateral walls and floor of maxillary sinus. There was also evidence of involvement of right eustachian tube, ethmoidal wall, and nasopalatine canal. An intraosseous malignancy of the palate was suspected, and a total maxillectomy was performed. The tissue sample was sent for histopathological assessment wherein changes diagnostic for polymorphous low-grade adenocarcinoma of the palate were observed.

**Conclusion:**

PAC is a distinct, yet commonly occurring, minor salivary gland tumor with varied clinical and histologic appearance. This case report highlights the importance of CBCT in diagnosing the intraosseous involvement of such tumors which can help shed some light in enhancing our knowledge about the minor salivary gland malignancies like PAC.

## 1. Background

Polymorphous low-grade adenocarcinoma (PLGA) is a slow growing malignant tumor of minor salivary glands and is generally of indolent nature. However, according to the most recent WHO Classification of Salivary Gland Tumors (2017), the cancer is classified as Polymorphous AdenoCarcinoma (PAC) [1]. Among all the tumors of oral salivary glands, oral salivary gland carcinomas comprise only 20% of the malignant neoplasms [2]. Lobular carcinoma, terminal duct carcinoma, and low-grade papillary adenocarcinoma were the various names that were used for adenocarcinomas of the minor salivary glands, before Evans and Batsakis [3] categorized them as a separate histologic entity in 1984. The World Health Organization later categorized PAC as a separate category of tumors of minor salivary glands in 1990 [4].

Due to its architecturally diverse structure, clinically indolent behavior, and cytological uniformity, PAC is thought to be a unique entity [5, 6]. Being asymptomatic with a low metastatic potential, it is commonly seen in elder women, with about 24% of primary tumors associated with major salivary glands (i.e., parotid, submandibular, or sublingual gland) and few in locations other than the head and neck, such as the breast and vagina [7, 8].

Although the third most commonly presenting malignant salivary gland tumor, our knowledge about PAC remains restricted to published case reports. Also, the architectural diversity associated with the name can be confusing for histopathological evaluation and can potentially be a diagnostic dilemma. Salivary gland neoplasms can be aggressive, and most of their clinical features often overlap; hence, a detailed examination and clinical investigation should be carried out in order to reach a definitive conclusion. Radiology is an important diagnostic tool for salivary gland pathology. It helps to determine the extent of involvement of bony structures, which can provide foresight into the treatment planning and provide a valuable tool in assessing the clinical picture. Cone Beam Computed Tomography (CBCT) is useful in the assessment of osseous structures and can help the clinician to determine the aggressive nature of the tumor as well as bony involvement due to a clear image of structures that are highly contrasted, and in comparison to most 2-D imaging methods, CBCT can provide a three-dimensional image of structures of the head and neck [9]. The following case report describes a unique case where a patient reported with the complaint of a soft-tissue growth in relation to the maxilla. This case report is aimed at highlighting the importance of CBCT in the detection of malignancies of salivary gland tissues, especially those cases with extensive bony involvement.

## 2. Case Presentation

**Figure 1:**
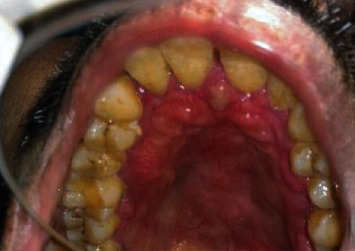
Evidence of diffuse, proliferative soft tissue swelling in the region of the hard palate.

**Figure 2:**
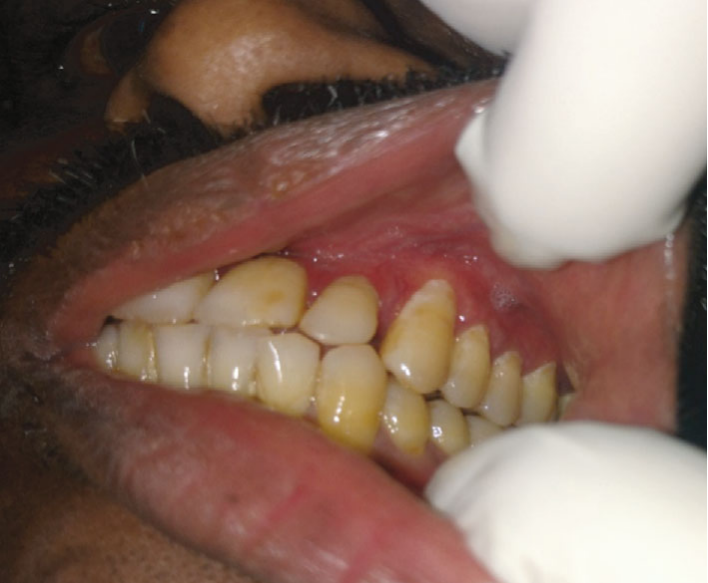
Evidence of erythema of the attached gingiva in relation to maxillary posterior teeth. The clinical features resembled a periodontal pathology.

A 47-year-old man was referred to the Department of Oral Medicine & Radiology with a history of soft tissue growth in the palate for the past two months. The growth was sudden in onset, growing rapidly in size over the next two months and localized to the palate. The patient reported no pain during the onset of growth. However, for the past one week, the patient reported mild pain. On clinical examination, there was a diffuse, proliferative soft tissue growth of the maxilla extending from the marginal gingiva in the palatal aspect of the anterior tooth and involving the entire surface of the hard palate up to the level of the hard and soft palate junction along with erythema of attached gingiva in relation to posterior teeth palatally and buccally as well as Grade III mobility of all the anterior and posterior maxillary teeth (Figures 1 and 2). The cervical group of lymph nodes were nonpalpable clinically.

**Figure 3:**
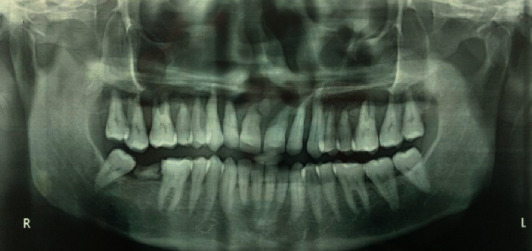
Panoramic radiograph of the patient revealing evidence of interdental bone loss in relation to all the maxillary teeth.

The referring private practitioner had advised a panoramic radiograph earlier which revealed severe bone loss in relation to maxillary teeth suggestive of generalized periodontitis and was inconclusive for any other relevant findings except for a root stump in the lower right quadrant (Figure 3).

**Figure 4:**
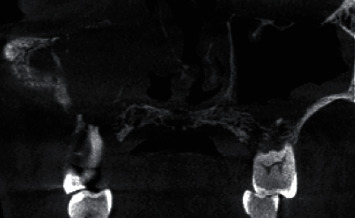
Coronal section of CBCT scan revealing destruction of walls of maxillary sinus along with involvement of nasopalatine canal.

**Figure 5:**
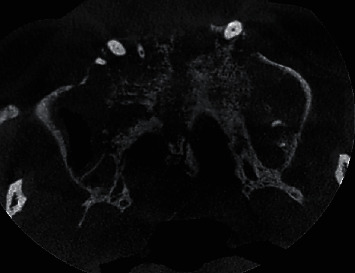
Axial view of CBCT scan revealing moth-eaten appearance of maxilla involving the hard palate and maxillary bone.

**Figure 6:**
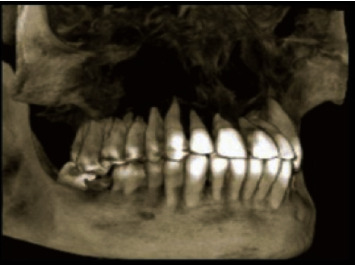
3D reconstructed CBCT image revealing widespread destruction of maxilla and resorption of all maxillary teeth root apices.

**Figure 7:**
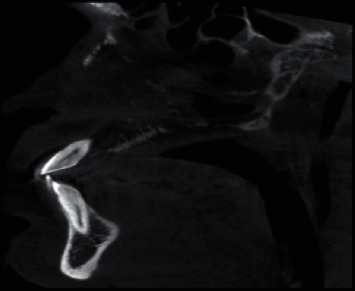
Sagittal section of CBCT revealing destruction of hard palate.

**Figure 8:**
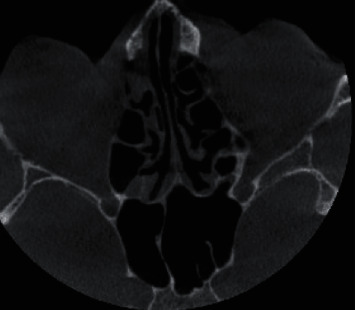
Axial view of CBCT scan revealing involvement of anterior and middle ethmoidal air sinus on the right side.

**Figure 9:**
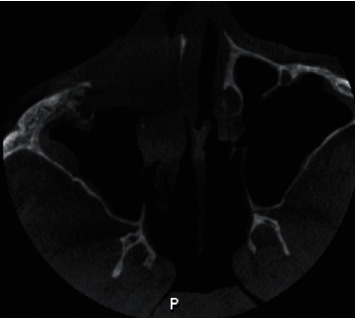
Axial view of CBCT scan revealing narrowing and involvement of eustachian tube on the right side.

The patient was advised for a CBCT scan which revealed a moth-eaten appearance of the palate noticed bilaterally with involvement of nasopalatine canal anteriorly and external resorption of roots in relation to all the maxillary teeth present, with evidence of involvement of anterior and middle ethmoidal sinus, eustachian tube, and nasopharynx on the right side. Based on CBCT and clinical findings, a preliminary diagnosis of intraosseous malignancy involving the palate was suspected (Figures 4–9). The patient was then scheduled for surgery with excision biopsy and total maxillectomy up to the floor of orbit.

**Figure 10:**
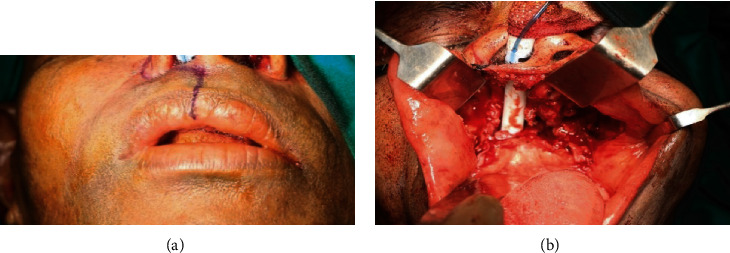
(a, b) Maxillectomy procedure done under local anesthesia.

**Figure 11:**
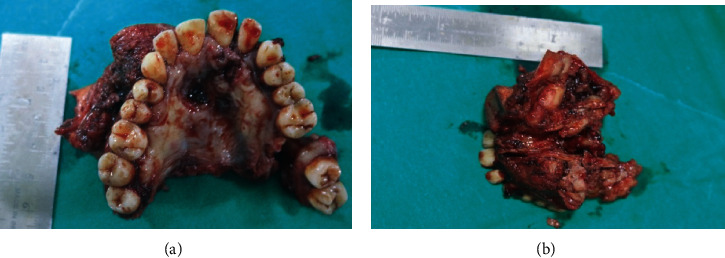
(a, b) Postsurgical maxillectomy specimen.

The surgical procedure consisted of visualization of hard and soft palate and incision extending laterally around the maxillary tuberosity exposing the posterior border. Following exposure of the maxillary tuberosity, attachment to the soft palate and hard palate was divided. After soft tissue dissection up to the extension of the tumor, the extent of the bone which was tailored to the primary tumor including the lateral wall of the orbit and zygoma to be resected was marked. Following bone guttering, the bony resection was carried out using a chisel and mallet through the frontal process of the maxilla and lacrimal bone. Bleeding encountered during this procedure from the greater palatine artery and the branches of internal maxillary artery was controlled using pressure packing followed by ligation to achieve haemostasis. After mobilizing the segment, final osteotomy was done to separate the maxillary tuberosity from the pterygoid plates. Before removal, thorough clinical examination of the specimen was done to determine the adequacy of the tumor (Figures 10(a) and 10(b) and 11(a) and 11(b)).

**Figure 12:**
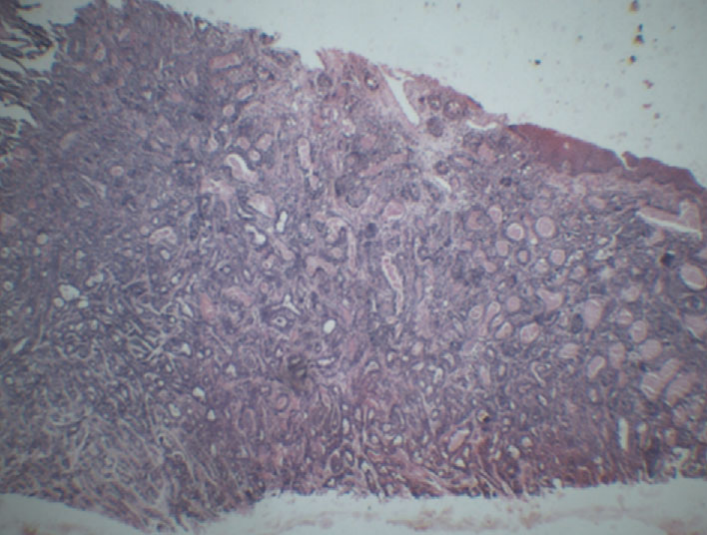
H and E section of tissue sample showing unencapsulated tumor close to surface epithelium with tumor cells arranged in solid nests.

**Figure 13:**
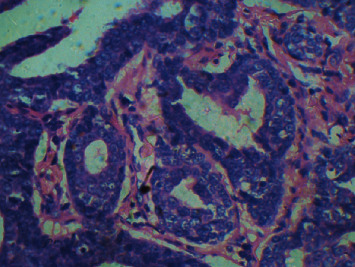
H and E sections showing ductal pattern of arrangement of tumor cells with evidence of vesicular nuclei.

**Figure 14:**
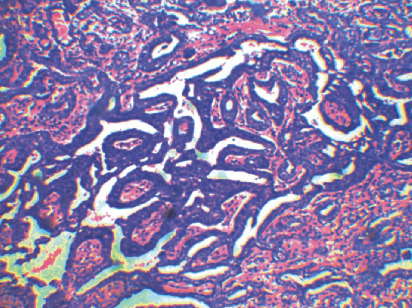
H and E sections showing arrangement of tumor cells in tubular pattern in the given tissue specimen.

The tissue sample was sent for histopathological examination which revealed an unencapsulated tumor within the underlying connective tissue approximating the surface epithelium, with a varied pattern of arrangement of tumor cells that included solid nests, strands, ducts, and tubular and papillary patterns and was lined by 1-2 layers of oval/cuboidal cells along with a peripheral layer of flat cells suggestive of polymorphous low-grade adenocarcinoma of the palate (Figures 12, 13, and 14). The patient is currently under follow-up for the past six months and is asymptomatic.

## 3. Discussion and Conclusion

Clinically, PAC presents as a firm, well-circumscribed, painless, and slow growing mass mostly covered by nonulcerated mucosa resembling a benign neoplasm and could be fixed to underlying structures, eroding and infiltrating the underlying bone, and is even present with perivascular and perineural invasion. Metastasis when reported is mostly confined to the regional nodes while spread to distant sites is a rarity and so is the transformation of this low-grade entity into a high-grade one [10].

Immunohistochemistry (IHC) has been utilized in a number of case series to distinguish between PAC and ACC, but there is a considerable overlap. Past studies conducted regarding the expression of ki-67 have consistently reported a very low labelling index (LI) in PAC which can assist in differentiating it from adenoid cystic carcinoma (ACC) [11–14]. In a few studies, p40 immunostaining has been demonstrated as a new tool for distinguishing salivary gland tumors with true myoepithelial differentiation from those showing nonspecific p63 expression. When these immunostains are performed in tandem, a discordant p63/p40-immunophenotype can reliably distinguish PAC from both adenoid cystic carcinoma and pleomorphic adenoma, which generally show p63/p40 concordance [15]. Genetic abnormalities may be useful in differential diagnosis of PAC. PACs harbor PRKD1 mutations in >70% cases, and PRKD1 mutations appear to be pathognomonic for this entity. In contrast, a vast majority of adenoid cystic carcinomas harbor MYB or MYBL1 gene rearrangements. While still not used widely in the clinic, use of these genetic markers may greatly facilitate the correct diagnosis [16].

The differential diagnosis for PAC includes pleomorphic adenoma and adenoid cystic carcinoma. PAC presents with a mucoid to the hyalinized matrix in comparison to pleomorphic adenoma which presents with a chondromyxoid matrix. Also, when compared to pleomorphic adenoma, PAC presents with perineural invasion. Another differentiating feature between the two is that PAC stains positive for S-100 and epithelial membrane antigen (EMA) whereas pleomorphic adenoma stains positive for glial fibrillary acidic protein (GFAP). When compared to adenoid cystic carcinoma, staining is positive for p-53, ki-67, bcl-2, and CD117. Perineural invasion is a feature of both PAC and adenoid cystic carcinoma, but the presence of the targetoid arrangement of perineural invasion is characteristic of PAC [17]. Also, in comparison to PAC, the cells of adenoid cystic carcinoma are smaller with hyperchromatic and angulated nuclei, less cytoplasm, and a coarser nuclear chromatin [18].

In the published literature, radiographic imaging of PAC has been mainly restricted to CT imaging since they provide a better soft-tissue contrast when compared to CBCT [19]. However, according to our knowledge, only a few case reports have highlighted the importance of CBCT in diagnosis of PGLA which describes it as presenting with no evidence of bone scalloping/erosion [20]. The radiographic appearance of PAC in our study resembled that of osteomyelitis and intraosseous malignancy. However, osteomyelitis of jaws is more common in the mandible when compared to the maxilla. Also, osteomyelitis of jaws is preceded most commonly with an identifiable cause of odontogenic infection such as a decayed tooth which was absent in our case. Intraosseous malignancies also present with a poorly defined, moth-eaten appearance. However, they occur more frequently in the mandibular posterior sections compared to the maxilla [21]. Also, these tumors reveal changes suggestive of carcinoma in the microscopic sections as defined by Suei et al. for a definitive diagnosis of intraoral malignancy.

Adjuvant radiotherapy has been indicated in cases where there is metastasis of cervical lymph nodes. A wide excision has been shown to minimize the rate of recurrence in cases of PAC, and radical excision has been suggested for the recurrent cases [22].

### 3.1. Summary

Minor salivary gland malignancy is an uncommon occurrence in daily practice. However, being the third most common minor salivary gland malignancy, it is necessary for the diagnostician to be well-versed with the clinical, histopathological, and radiographic features of PGLA. Although the knowledge available on this entity is limited, it is necessary to be able to segregate it from the miscellaneous tumors of minor salivary glands in order to provide appropriate treatment for the patient. Due to its tendency for recurrence, it is important to regularly follow-up the patient posttreatment. The importance of radiography especially 3D imaging in the form of CBCT in dental practice is highlighted in this case report as we have noticed that even though salivary gland malignancies usually limit themselves to soft tissue involvement, there are cases wherein extensive involvement of bone with a varied radiological presentation can be observed.

PAC is a distinct, yet commonly occurring, minor salivary gland tumor with varied clinical and histologic appearance. This case report highlights the importance of CBCT in diagnosing the intraosseous involvement of such tumors which can help shed some light in enhancing our knowledge about the minor salivary gland malignancies like PAC.

## Data Availability

The datasets generated and/or analysed during the current study are not publicly available for the protection of privacy of the patient but are available from the corresponding author on request.

## Abbreviations

PAC: Polymorphous low-grade adenocarcinoma
CT: Computed tomography
CBCT: Cone beam computed tomography
ACC: Adenoid cystic carcinoma
PA: Pleomorphic adenoma
IHC: Immunohistochemistry
LI: Labelling index
EMA: Epithelial membrane antigen
GFAP: Glial fibrillary acidic protein.
